# Who Holds Up the World?

**Published:** 2007-03-15

**Authors:** Lynne S. Wilcox

This issue of *Preventing Chronic Disease* (*PCD*) is devoted to global health and explores the efforts of several countries to respond to the challenges of chronic disease and support the health and well-being of their citizens. From earliest human history, people have created myths that depict the sacred and at times terrible responsibility of supporting the world. Although these myths vary from culture to culture ― and the entities charged with the awesome responsibility of holding up the earth range from deities to animals to the elements ― the underlying purpose of all of them is to assure people of the world's stability and order. In the Haudenosaunee (i.e., Six Nations or Iroquois) (1,2), Hindu (3), and Gabrielino Indian (4) religions, turtles and tortoises support the earth. The indigenous Japanese Ainu people describe the world as a vast ocean resting on the backbone of a trout that creates the surging of the tides each day by sucking in the ocean and spewing it out (5). In other mythologies, a single entity is responsible for carrying the heavy burden of the world. In Greek mythology, for example, Atlas was forced to support the earth after fighting unsuccessfully against Zeus, the leader of the Olympian gods. Hercules came to Atlas and requested that he obtain the Hesperides' golden apples. Atlas agreed on the condition that Hercules would support the earth while he was away. Atlas had no intention of accepting his eternal burden again, but Hercules tricked him into taking it back (6).

Fortunately, countries throughout the world today are approaching their responsibility to support their citizens' health more willingly than Atlas approached his task. In more recent years they have increasingly supported efforts to address the growing worldwide burden of chronic diseases such as cancer, diabetes, cardiovascular disease, and chronic respiratory illness. The World Health Organization (WHO) estimates that chronic disease accounts for 35 million deaths per year, 80% of which occur in low- to middle-income countries (7). Countries that lose their citizens to early deaths from chronic diseases face billions of dollars in reduced productivity.

In 2005, WHO published *Preventing Chronic Diseases: A Vital Investment* to promote the health of the earth's people (7). In it, WHO recommends that efforts to reduce the prevalence and impact of chronic disease include three steps (i.e., the STEPwise Program) to promote the health of the earth's people (7). In it, WHO recommends that efforts to reduce the prevalence and impact of chronic disease include three steps (i.e., the STEPwise Program): 1) estimating population needs, 2) developing a health policy, and 3) implementing programs (8). Use of the STEPwise approach to support people's health is illustrated in this issue of *PCD* by Minh et al, who describe a survey in Vietnam, designed on the basis of WHO's surveillance recommendations, that was used to gather data on the prevalence of risk factors for chronic disease in a rural population (9).

Research and surveillance are also important in developing health policy, as McDonald demonstrates in examining a program's efforts to use newspaper advertisements to recruit participants into healthy-eating activities in Ontario (10). This issue also contains two articles that describe efforts to assess the health of two U.S. immigrant groups ― Mexican Americans with diabetes (11) and Filipino children (12) ― and another article that compares the relative effectiveness of the antitobacco efforts of two countries by comparing the responses of U.S. youths to American cigarette warning labels and announcements with their responses to warning labels and announcements used in Canada (13).

The metaphor of supporting the earth is also reflected in the myths of cultures that consider holding up the earth or the sky to be a shared responsibility. According to the ancient Norse religion, four dwarfs — Austri, Vestri, Sudri, and Nordri — held up the four corners of the earth (14), while the Mayans believed that four gods, the Bacabs, supported the sky (15). Several articles in this issue describe similar cooperative efforts to support the health of a community. Robinson et al describe the approach of seven Canadian provinces in disseminating information for the Canadian Heart Health Initiative and their eventual decision to provide the public with a wide variety of information about how to prevent chronic disease and promote healthy living (16). Coalition and partnership building, policy advocacy, and strategy development were key elements in the projects they describe. Kunyk et al describe how Capital Health, a provincial health authority in Edmonton, Alberta (17), adopted a smoke-free environment at all of its facilities. Managers, policy makers, and frontline health care professionals were all involved in designing educational programs for staff members and in designing protocols for providing nicotine replacement therapy to staff members as well as patients.

Finally, public health professionals concerned with international health may find inspiration in mythological stories that describe the role of the elements in supporting the earth. In ancient Egyptian mythology, Shu, god of the air, separated the sky (his daughter Nut) from the earth (his son Geb) (18). In Shintoism, it is the god of wind, Shine-Tsu-Hiko, who holds up the sky (19). As countries throughout the world continue to develop the knowledge, skills, and systems to support the health of their citizens, we who address the global impact of chronic diseases can assist them by providing some of the elements vital to the success of their developing health care systems.
